# Genetic engineering of baculovirus-insect cell system to improve protein production

**DOI:** 10.3389/fbioe.2022.994743

**Published:** 2022-09-20

**Authors:** Minqing Hong, Tingting Li, Wenhui Xue, Sibo Zhang, Lingyan Cui, Hong Wang, Yuyun Zhang, Lizhi Zhou, Ying Gu, Ningshao Xia, Shaowei Li

**Affiliations:** ^1^ State Key Laboratory of Molecular Vaccinology and Molecular Diagnostics, School of Life Sciences, School of Public Health, Xiamen University, Xiamen, China; ^2^ National Institute of Diagnostics and Vaccine Development in Infectious Diseases, Xiamen University, Xiamen, China; ^3^ Xiang An Biomedicine Laboratory, Xiamen, China; ^4^ The Research Unit of Frontier Technology of Structural Vaccinology of Chinese Academy of Medical Sciences, Xiamen, China

**Keywords:** baculovirus, insect cell, genetic engineering, protein production, glycosylation

## Abstract

The Baculovirus Expression Vector System (BEVS), a mature foreign protein expression platform, has been available for decades, and has been effectively used in vaccine production, gene therapy, and a host of other applications. To date, eleven BEVS-derived products have been approved for use, including four human vaccines [Cervarix against cervical cancer caused by human papillomavirus (HPV), Flublok and Flublok Quadrivalent against seasonal influenza, Nuvaxovid/Covovax against COVID-19], two human therapeutics [Provenge against prostate cancer and Glybera against hereditary lipoprotein lipase deficiency (LPLD)] and five veterinary vaccines (Porcilis Pesti, BAYOVAC CSF E2, Circumvent PCV, Ingelvac CircoFLEX and Porcilis PCV). The BEVS has many advantages, including high safety, ease of operation and adaptable for serum-free culture. It also produces properly folded proteins with correct post-translational modifications, and can accommodate multi-gene– or large gene insertions. However, there remain some challenges with this system, including unstable expression and reduced levels of protein glycosylation. As the demand for biotechnology increases, there has been a concomitant effort into optimizing yield, stability and protein glycosylation through genetic engineering and the manipulation of baculovirus vector and host cells. In this review, we summarize the strategies and technological advances of BEVS in recent years and explore how this will be used to inform the further development and application of this system.

## Introduction

The Baculovirus expression vector system (BEVS) was established in the 1980s when it was first used as a recombinant baculovirus to produce heterologous human IFN-β protein in insect cells (Mishra, 2020). Since then, thousands of heterologous proteins, virus-like particles, surface-displayed protein/antigen vectors, heterologous viral vectors, and gene delivery vectors have been produced using this system, including several licensed protein-based human and veterinary vaccines, and the first approved gene therapy product (BEVS-derived recombinant adeno-associated viruses) in the western world (Mishra, 2020). Nowadays, the continuous improvement in BEVS has increased its use as solely a research tool to a mature manufacturing platform for new biologics.

The BEVS platform is based on the natural propensity of baculoviruses to infect insect cells. More than 600 baculoviruses have been isolated in nature. Their host-specificity is usually restricted to a single or a few insect species. The BEVS platform encompasses three core parts: Baculovirus production, insect cell infection, and purification. First, recombinant baculovirus (rBV) carrying foreign genes were produced by the recombination or transposition method. Next, the rBV is then amplified via several rounds of passaging to create a high titer of rBV stock, which is then used to infect insect cells with an optimized multiplicity of infection (MOI) to produce the target protein. Linearized baculovirus DNA leads to significantly improved recombination efficiency and reduces the tedious work of screening for positive recombinant viruses using plaque assays (Felberbaum, 2015; Martinez and Targovnik, 2019). Three days after infection, cells and/or the supernatant are collected and purified based on standard purification technologies to obtain the protein of interest.

In this review, we have recapped the advanced applications of BEVS in the biomedical research, including its application in the development of vaccines against COVID-19 and other infectious diseases, and the advantages, limitations and improvement considerations of BEVS, then summarized the fundamental research and major technological advances on the baculovirus vectors, genetic engineering of viral genome composition and insect cell lines to overcome the limitations on protein expression levels, instability and lower-extend glycosylation profiles. This review blueprints challenge and improvement of the versatile BEVS, and will benefit the researchers in this area.

### The products based on baculovirus expression vector system

The BEVS has been extensively used in biomedicine for vaccine production, stem cell transduction, tissue engineering, viral vector production, gene therapy, cancer therapy, and other areas (Gomez et al., 2014; Rong et al., 2019). To date, eleven BEVS products have been approved, including four human vaccines (Cervarix, Flublok, Flublok Quadrivalent, and Nuvaxovid/Covovax), two human therapeutics (Provenge and Glybera), and five veterinary vaccines (Porcilis Pesti, BAYOVAC CSF E2, Circumvent PCV, Ingelvac CircoFLEX and Porcilis PCV) (Felberbaum, 2015), (Table 1). Cervarix was the first human BEVS-related vaccine approved by the US Food and Drug Administration (FDA) in 2009 for the prevention of cervical cancer. Cervarix contains bivalent virus-like particles (VLPs) comprising human papillomavirus (HPV) L1 proteins for HPV types 16 and 18. Later, in 2013, FluBlok was approved, the first recombinant protein vaccine in the prevention of seasonal influenza. Flublok and the Flublok Quadrivalent contain three or four types of recombinant hemagglutinin (HA) antigens from influenza A- and B-type viruses. During the 2014–2015 influenza season in the United States, the incidence of flu among those treated with FluBlok Quadrivalent vaccine was 30% lower than that in the inactivated vaccine group [Fluarix Quadrivalent (Cox et al., 2008; Dunkle et al., 2017a; Dunkle et al., 2017b)].

**TABLE 1:** A summary of typical products based on BEVS.

	Target	Antigen	Band name	Manufacture	Stage	References
Human vaccines	Influenza virus	HA protein	FluBlok	Sanofi Pasteur	Approved	Cox et al. (2008)
	Influenza virus	HA protein	Flublok Quadrivalent	Sanofi Pasteur	Approved	Dunkle et al. (2017b)
	Human papillomavirus	HPV16/18 L1 protein	Cervarix	GSK	Approved	Paavonen et al. (2007)
	COVID-19	Spike (S) protein	Nuvaxovid/Covovax	Novavax	Approved	Guebre-Xabier et al. (2020)
Human therapeutics	Prostate cancer	PAP-GM-CSF fusion protein	Provenge	Dendreon	Approved	Cheever and Higano, (2011)
	Hereditary lipoprotein lipase deficiency (LPLD)	AAV1 viral vector with an intact copy of the human lipoprotein lipase (LPL)	Glybera	uniQure	Approved	Steinhagen-Thiessen et al. (2017)
Animal vaccine	Classical swine fever	E2 protein	Porcilis Pesti	MSD Animal Health	Approved	van Oirschot, (2003)
	Classical swine fever	E2 protein	BAYOVAC CSF E2	Bayer AG/Pfizer Animal Health	Approved	Lai et al. (2019)
	Porcine circovirus-2	PCV2 ORF2 protein	CircoFLEX	B. Ingelheim	Approved	Fachinger et al. (2008)
	Porcine circovirus-2	PCV2a Cap protein	Cirumvent PCV	Merck Animal Health	Approved	Segales, (2015)
	Porcine circovirus-2	PCV2 ORF2 protein	Porcilis PCV	MSD Animal Health	Approved	Blanchard et al. (2003)
Human vaccines	COVID-19	recombinant RBD monomer	—	West China Hospital of Sichuan University	Phase III	Yang et al. (2020)
	COVID-19	CoV2 preS dTM	—	Sanofi/GSK	Phase III	Pavot et al. (2022)
	Influenza A H1N1	A (H1N1) 2009 Influenza Virus-like Particle		Novavax	Phase II	Lopez-Macias et al. (2011)
	Seasonal Influenza virus	Hemagglutinin (HA)、 neuraminidase (NA) and matrix 1 (M1)	Nanoflu	Novavax	Phase III	Smith et al. (2017)
	Human parvovirus B19	VP1 and VP2	—	National Institute of Allergy and Infectious Diseases/Meridian Life Science	Phase I/II	Bernstein et al. (2011)
	Norwalk virus	Norwalk virus-VLP	—	Baylor College of Medicine	Phase II	Atmar et al. (2011)
	Norwalk virus	Norwalk virus-VLP	—	Ligocyte	Phase I	ElKamary et al. (2010)
	Respiratory syncytial virus	Fusion glycoprotein	—	Novavax	Phase III	Fries et al. (2017)
	COVID-19 and Influenza	Quadrivalent Hemagglutinin Nanoparticle Influenza and SARS-CoV-2 rS Nanoparticle		Novavax	Phase I/II	
	Ebola	Ebola Virus (EBOV) Glycoprotein (GP)	—	Novavax	Phase I	Bengtsson et al. (2016)
	Malaria	ChAd63ME-TRAP/MVA ME-TRAP Heterologous	—	Novavax	Phase I	Venkatraman et al. (2017)
	Human papillomavirus	HPV (6/11/16/18/31/33/35/39/45/51/52/56/58/59) L1 protein	—	SinoCellTech	Phase I	
Human therapeutics	Type I diabetes	Glutamate decarboxylase (GAD)	Diamyd	Diamyd	Phase III	Beam et al. (2017)

Nuvaxovid/Covovax is one of the more recent BEVS-derived vaccines authorized by European Medicine AGENCY (EMA, in 2021), India (in 2021) in Australia (in 2022), WHO emergency use listing (EUL, in 2021), Health Canada (in 2022) and FDA (in 2022) for the prevention of coronavirus disease 2019 (COVID-19) in people aged 18 years and older. In phase 3 clinical trials in the United States and Mexico, the vaccine was 90% effective in preventing mild, moderate, and severe cases of COVID-19 during a pandemic of multiple variants (Alpha, Beta, and Delta) (Guebre-Xabier et al., 2020). Another COVID-19 vaccine based on BEVS developed by West China Hospital of Sichuan University is in phase 3 clinical trials (Possee et al., 2020; Yang et al., 2020). Sanofi-GSK also developed a COVID-19 vaccine candidate: an adjuvanted bivalent D614G and Beta (B.1.135 variant) vaccine that contains the antigen CoV2 preS dTM produced by BEVS (Pavot et al., 2022). According to the results of a recent phase 3 clinical trial, the Beta-containing vaccine offered 64.7% efficacy against symptomatic infection in adults, 75.1% efficacy in participants previously infected with COVID-19, and 72% efficacy against Omicron. This efficacy data, reported for the first time in an Omicron environment, supports the relevance of a Beta-containing vaccine candidate (https://www.sanofi.com/en/media-room/press-releases/2022/2022-06-24-05-29-02-2468538).

In 2012, the EMA first approved Glybera, a gene therapy product for the treatment of hereditary lipoprotein lipase deficiency (LPLD). Glybera is composed of an adeno-associated virus serotype 1 (AAV1) viral vector with an intact copy of the human lipoprotein lipase (LPL) gene manufactured in BEVS (Steinhagen-Thiessen et al., 2017). Prior to this, in 2010, the FDA approved the use of Provenge (Sipuleucel-T), an autologous immunotherapy medicine, for the treatment of prostate cancer. In this therapy, the patient’s dendritic cells are first isolated and then treated by pulsing with a PAP-GM-CSF fusion protein derived from BEVS-produced prostatic acid phosphatase (Cheever and Higano, 2011). The modified dendritic cells are then administered back to the patient. Other vaccines produced using BEVS, such as the vaccine against H7N9 avian influenza virus (Li et al., 2021) or recombinant ferritin to display foreign proteins (Qu et al., 2020) are in clinical trials or preclinical studies, respectively, at the time of writing. In addition, BEVS is being used for the production of diagnostic antigens (Ebihara et al., 2021) encountered as a consequence of emerging infectious diseases.).

### The advantages and limitations of baculovirus expression vector system

There are many advantages of BEVS. First, the BEVS platform has inherent security measures that are attractive from a regulatory perspective. Baculoviruses only infect insect cells not other vertebrates (Shokrollahi et al., 2021); indeed, in safety tests conducted by the European Commission’s Health and Consumer Protection Directorate-General in 2008, there were no adverse effects of baculoviruses on human health and no pathogenic, carcinogenic, or genotoxic changes to mammalian cells (Premanand et al., 2018). In addition, baculoviruses can neither replicate within transduced cells nor integrate their DNA into the host genome without selective pressure (Lu et al., 2012). Second, the baculovirus genome can accommodate multi-gene– or large gene insertions while also allowing for post-translational modifications that are essential for proper protein function. For instance, BEVS is suitable when trying to express complex or difficult-to-express proteins, such as various enzymes, parasite proteins (Gomez et al., 2014), glycoproteins, G protein-coupled receptors (GPCRs), virus-like particles (VLPs), and protein machines, among others (Dias et al., 2021). In a head-to-head comparison study, BEVS led to better protein expression in High five insect cells than did the Expi293F human cell system, suggesting that the High five cell system is particularly suitable for producing difficult-to-express proteins (Korn et al., 2020). BEVS is also widely used for functional, crystallographic, and drug discovery studies (Sari et al., 2016; Pena and Kamen, 2018), as it offers the unique advantage of producing proteins bearing appropriate post-translational modifications. Third, insect cells are grown in suspension in serum-free medium, which allows for an easy scale-up production. Indeed, express SF+ cells an produce recombinant proteins at scales ranging from 2 to 21,000 L.

A single insect cell line can be used for numerous products, particularly for single-use or single-use technologies that require rapid production. Indeed, a single BEVS facility can rapidly produce vaccines against an epidemic or pandemic, eliminating the steps required in screening for stable cells. Furthermore, compared with traditional inactivated vaccine platforms, there is no requirement for expensive biocontainment because the BEVS platform does not need to deal with live or potentially dangerous pathogens. Given that numerous BEVS-derived products have been approved, the required regulations are now in place. The advantages of proper protein folding and post-translational modification, serum-free culture, and scalability to large capacity all make it attractive in future biological applications (Felberbaum, 2015; Rong et al., 2019).

Although many proteins have been successfully expressed with this platform, some limitations and considerations need to be addressed in future studies. For example, the cytopathic effects caused by viral evasion resulting in lysis of host cell impact the final product yield compared with mammalian expression; the N-glycosylation profile of heterologous protein produced in insect cells differs from that produced by mammalian protein. In addition, BEVS induces an immune response that produces inflammatory cytokines and chemokines and activates the complement pathway (Betenbaugh et al., 1996). This, in turn, can lead to viral genome degradation, suppressed transgene expression, unstable protein expression and baculovirus genome instability (Grose et al., 2021). Any of these issues may limit the further development of BEVS. However, the BEVS platform has been greatly improved in recent years, with evidence of vector optimization, improvements in viral genome modifications, and extensive applications of host cell engineering. These molecular advances help to facilitate the versatility of the BEVS platform for biologics development (Rong et al., 2019). Specifically, two primary components associated with BEVS—baculovirus vector and insect cells—have been improved in recent years, as discussed in the next section.

## Engineering of baculovirus vector

### Development of baculovirus vectors

Baculoviruses, with a closed double-stranded circular DNA genome of 80–180 kbp, belong to the family *Baculoviridae*, which infects arthropods with high host specificity. Two types of baculoviruses are most used in studies: *Autographa californica multicapsid nucleopolyhedrovirus* (AcMNPV) and *Bombyx mori nucleopolyhedrovirus* (BmNPV). Although genetically close and share about 90% amino acid sequence identity within the open reading frame, these two types have a minimal overlap in host range, with AcMNPV having a broader host range than BmNPV (Liu et al., 2021). AcMNPV was the first baculovirus to be sequenced in its entirety and is thus considered a baculovirus study model (Bai et al., 2020).

Various regions of the baculovirus genome have been sequenced, and indicate that AcMNPV might only encode 150 genes. AcMNPV gene expression is divided into four stages: Very early expression, early expression, late expression, and very late expression. The BEVS introduces foreign genes into non-essential regions of the viral genome through homologous recombination and uses polyhedrin (*polh/pH*) or *p10* promoters to produce recombinant proteins in insect cells (Bleckmann et al., 2016; Martínez-Solís et al., 2016; Kong et al., 2018). *pH* and *p10* are two very late genes that produce highly expressed proteins in the very late stage, and their gene promoters have a strong activating ability (Kong et al., 2018; Rong et al., 2019; Liu et al., 2021). Polyhedrin is the main component that forms inclusion bodies, and it can accumulate in cells up to 50% at the later stage of infection. It is a non-essential component for virus replication. The *p10* protein is also a non-essential component of virus replication and might be associated with cell lysis.

During the infection process, two virus particles are produced: budded viruses (BVs) and occlusion-derived virus particles (ODVs). BVs and ODVs have the same nucleocapsid but different envelopes. BVs consist of a single nucleocapsid surrounded by an envelope which are responsible for the horizontal transmission from cell to cell whereas ODVs contain either single or multiple nucleocapsids which are responsible for the horizontal transmission between insects (Rong et al., 2019). Baculoviruses vector modification revolutionized the BEVS technology and eliminated the need for tedious ODV-based selection. Various baculovirus vectors have been constructed and optimized for use, for instance, inserted restriction site *Bsu36* I in *lacZ* to linearize baculovirus DNA, co-transfecting the transfer vector into insect cells, only after the homologous recombination of baculovirus DNA and transfer vector can form a complete DNA and generate baculovirus to infect insect cells, which greatly improves recombination frequency of the baculovirus and the transfer plasmid carrying the foreign gene. The addition of two *Bsu36* I cutting sites in *orf1629* and *orf603* linearized and deleted essential portions of the *orf1629*. Co-transfecting with a transfer vector rescued the lethally deleted virus and led to recombination frequencies of 99% (vector including BaculoGold, BestBac) (Felberbaum, 2015; Martinez and Targovnik, 2019). Other circular DNA has a part of *orf1629* genes deletion and contains bacterial artificial chromosome (BAC). Homologous recombination can then restore *orf1629* to produce a recombinant baculovirus (vectors such as BacMagic, BacPAK/flashBAC). Another commercial system, Bac-to-Bac, generates recombinant baculoviruses by site-specific transposition in *E. coli* (DH10bac) rather than homologous recombination in insect cells. Competent DH10Bac contains a parent bacmid with a lacZ-mini-attTn7 fusion, which promotes transposition between the vector and the bacmid. Its successful expression then abolishes the *lacZ* gene (Yang and Pang, 2003). Further modifications to the baculovirus backbone have allowed for the production of more complex and highly processed proteins, such as membrane and cytoplasmic proteins and those involved in protein machinery. Another commercially available BEVS, like BaculoDirect, uses Gateway Technology based on the bacteriophage lambda site-specific recombination system, which facilitates integration (Mishra, 2020).

### Promoter and enhancer optimization

**FIGURE 1:**
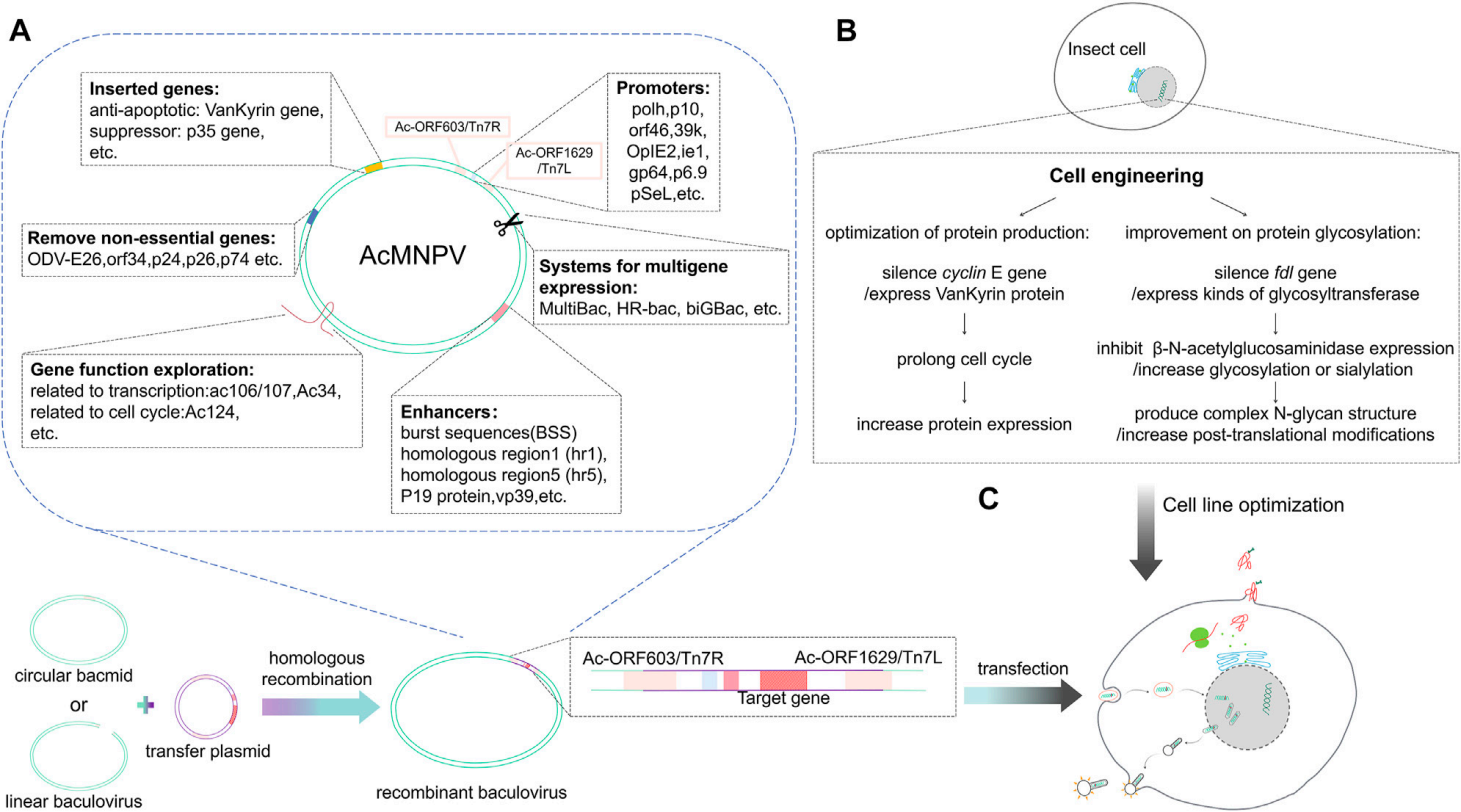
Baculovirus genome editing and the expression of foreign proteins. **(A)** The circular bacmid or linear baculovirus recombines with the transfer plasmid carrying the foreign gene through the T7 transposon or homology arm to form a recombinant baculovirus. The baculovirus is optimized at the molecular level, removing non-essential genes and inserting elements that are conducive to protein expression. After exploring gene functions of baculoviruses, efficient promoters and enhancers are selected to design transfer vectors that can carry foreign genes. **(B)** Insect cell line optimization mainly includes optimization of exogenous protein glycosylation and production. **(C)** The recombinant baculovirus infects insect cells; after entering the cells, the foreign protein is expressed under the transcriptional regulation of the insect cell.

To improve protein expression levels, essential genetic elements of the baculovirus vector can be optimized, including the promoter and the enhancer (Figure 1). In 2016, Martínez-Solís *et al.* showed that the *orf46* promoter offered high activity and, when used in conjunction with the *pH* or *p10* promoter, had an additive effect in expression (Martínez-Solís et al., 2016). The immediate early viral promoter, *OpIE2* (isolated from *Orygia pseudotsugata* multicapsid nucleopolyhedrovirus, OpMNPV), offers the highest activity in plasmid-based expression (Bleckmann et al., 2016). Mika Masumoto *et al.* reported the expression pattern of the immediate early IE1 promoter, and showed that it could effectively initiate the expression of foreign genes with piggy Bac-based vectors (Mika et al., 2012). Other earlier promoters like *39k* or *gp64* also contribute to the high expression of recombinant proteins in the early stages of cell infection (Martinez and Targovnik, 2019). In the transactivation system, protein expression can be boosted by co-infection of the plasmid-based expression and target-gene-free baculoviruses, homologous region 5 region (hr5), and immediate-early or very late viral promoters (*IE1* or *OpIE2* or *p10*) (hr5-IE1-p10 or hr5-OpIE2-p10 cassette) (Bleckmann et al., 2016; Martínez-Solís et al., 2016). The signal peptides also play an essential role in secreted protein expression. Sugai *et al.* found that introduction of the polar amino acid Asparagine into the C-terminus of the SP1 region could enhance the secretion of recombinant protein in the silkworm; this method can also be applied to other cell lines, e.g., *BmN* cell line, *Sf* cell line, and *Tn* cells (Sugai and Tsumoto, 2010) (Table 2).

**TABLE 2:** A summary of function studies related to BEVS presented in this review.

Element type	Technology	Element name	Result of study	References
Promoter	Molecular Cloning	OpIE2	Protein expression +	Bleckmann et al. (2016)
	Molecular Cloning	ie1	Protein expression +	Mika et al. (2012)
	Molecular Cloning/RNAi	gp64	Protein expression ++	Martinez and Targovnik, (2019)
	Molecular Cloning	39k	Protein expression +, Carbohydrase activity/Human N- Glycan ++	Martinez and Targovnik, (2019)
	Molecular Cloning	p6.9	Protein expression +	Martinez and Targovnik, (2019)
	Molecular Cloning	pSeL	Protein expression ++	Martinez and Targovnik, (2019)
Enhancer	Molecular Cloning	hr1	Protein expression ++	Tiwari et al. (2010)
	Molecular Cloning/CRISPR/Cas9	vp39	Late promoter transcription +, protein expression ++	Bai et al., (2019); Bai et al., (2020)
Promoter paired with enhancer	Molecular Cloning	ph/p10 + orf46	Protein expression ++	Martínez-Solís et al. (2016)
	Molecular Cloning	hr5+IE1+p10/hr5+OpIE2+p10	Protein expression ++	Bleckmann et al. (2016); Martínez-Solís et al. (2016)
	Molecular Cloning	vp39 + BSS+ph	Protein expression +++	Lee et al. (2018)
	Molecular Cloning	hr5+ph	Protein expression ++	Lee et al. (2018)
Signal peptide (SP)	Molecular Cloning	Introduce polar amino acid Asparagine into the C-terminus of SP1 region	Protein expression ++	Sugai and Tsumoto, (2010)
Other non-essential gene	CRISPR/Cas9	Delete p10/p24/p26/p74	Protein expression ++	
protein expression related gene	Molecular Cloning	Express P19 protein	Protein expression +++	Liu et al. (2015)
	RNAi	Silence pkip gene	P6.9 transcription -	Lai et al. (2020)
	RNAi	Silence ODV-E26 gene	Protein expression ++	IbarraI et al. (2019)
	RNAi	Silence orf34 gene	Protein expression ++	Salem et al. (2013)
	CRISPR/Cas9	Overexpression of ac34	38k/vp39 transcription +, protein expression+	Fang et al., (2019); Zhang et al., (2019)
	RNAi/CRISPR/Cas9	Silence FDL gene	n-glycans ++	Mabashi and Jarvis, (2017); Pena and Kamen, (2018)
	CRISPR/Cas9	Hsp40/Hsc70 human molecular chaperone	Protein folding ++	Yokoyama et al., (2000); Ngosuwan et al., (2003)
	Cre-LoxP fusion or Tn7R/L transposition	Multibac, HR-bac, biGBac	Multigene expression ++	Bieniossek et al., (2012); Kolesnikova et al., (2022); Weissmann et al., (2016)
Apoptosis related gene	Molecular Cloning	Expression of p35	Protein expression ++	Karamipour et al. (2018).
	CRISPR/Cas9	Expression of Vankyrin	Protein expression ++	Goodin et al. (2006)
	Molecular Cloning/CRISPR/Cas9	Delete ChiA/v-cath gene	Protein expression ++	Rao et al. (2004)
	RNAi/CRISPR/Cas9	Interfere caspase-1 gene transcribe	Inhibit apoptosis, protein expression ++	Ou and Xv, (2016); Rong et al., (2019)
	CRISPR/Cas9	Overexpression of ac124	Chitinase expression ++,	Fang et al., (2019); Zhang et al., (2019)
	RNAi	Silence cyclin E gene	Protein expression ++	Martinez and Targovnik, (2019); Pena and Kamen, (2018); Xiao et al., (2014)
Viral infection related gene	RNAi	Interfere Dcr2/Ago2 gene transcribe	Baculovirus infects insect cells ++	Karamipour et al. (2018)
	CRISPR/Cas9	ac106/107	Affect virus replication	Wang et al. (2021)
	CRISPR/Cas9	PSL1180-Cas9-sgIE1-sgLEF11-sgGP64 (sgMultiple)	Affect virus replication	Dong et al. (2019)

“+” means this optimization is effective, “++” means much better, “+++” means it has a large improvement.

In 2018, Lee et al. (2018) compared the effects of enhancers including hr5, burst sequences (BSS), and vp39 on *pH* activation ability (Figure 1). They showed that vp39 combined with BSS has the best enhancement effect on *pH* activation; hr5 comes second. This combination is effective in both AcMNPV and BmNPV. Another study found that mRNA transcripts and *pH* or *p10* promoter activity decreased following knockout of *vp39*, indicating that *vp39* contributes to activating late promoters (Bai et al., 2020). In another study by Bai *et al.*, deletion of AcMNPV *vp39* gene led to a defection in nucleocapsid assembly (Bai et al., 2019). Another late promoter, *p6.9*, activates the expression of different recombinant proteins; albeit, this is not as widely used as the other promoters. As an alternative to the *pH*, *pSeL*, derived from SeMNPV, can potentially increase the expression of recombinant proteins in different insect cell lines (Martinez and Targovnik, 2019). Tiwari et al. (2010) found a 3–4 times increase in protein expression with the homologous region 1 region (hr1) than without hr1 in both Sf21 and Tni insect cells. P19 protein, an RNAi inhibitor derived from *tombusvirus*, can act as an enhancer to significantly increase baculovirus production and gene expression downstream of the *pH* promoter or OpIE2 promoter in Sf9 cells (Liu et al., 2015) (Table 2.

### RNAi for improved protein expression

Baculovirus genetic engineering has benefitted from RNA interference (RNAi) and gene editing technologies that have emerged in recent years. RNAi, developed for gene manipulation and disease treatment, is a powerful tool that can specifically silence target gene expression using highly homologous double-stranded RNA (dsRNA) (Liao and Tang, 2016). Ou *et al.* constructed the dsRNA sequence of Sf-caspase-1 full-length on pBac5 and co-transfected Sf9 cells with AcMNPV Bacmid to obtain recombinant baculovirus vAcMNPV-dsCasp. The authors were able to reduce Sf-caspase-1 mRNA levels and inhibit cell apoptosis after infecting insect cells (Ou and Xv, 2016). Others have shown that interfering with *gp64* gene via small interfering RNA (siRNA) or dsRNA can reduce residual baculovirus contaminants and increase recombinant protein production by 30% (Pena and Kamen, 2018). IbarraI et al. (2019) showed that silencing ODV-E26, a non-essential gene for survival or infectivity, can increase recombinant protein production. Salem et al. (2013) showed that silencing the *orf34* gene (a transcription unit known as unfunctional but essential for baculovirus transmission) with RNAi enhances the expression of heterologous genes. Lai et al. (2020) used RNAi to silence *pkip* and showed that its deletion leads to the reduced hyperphosphorylation of P6.9, and the subsequent down-regulation of the transcription and hyperexpression of very late genes (Figure 1).

### Baculoviral genome editing

Genome editing has many applications. First, gene deletion is a valuable strategy to produce modified baculoviruses for improved recombinant protein production. In the late stages of baculovirus infection, insect cell secretion pathways are disrupted, limiting the extent to which the secreted recombinant proteins can be folded and secreted into the extracellular medium. Such secretion pathway impairment is caused partly by the large accumulation of virus-encoded chitinase (*chiA*), which works with a protease cathepsin [*v-cath*) and induces characteristic terminal liquefaction of the insect host in the final stages of the infection process (Rao et al., 2004)]; cathepsin may itself also be involved in the breakdown of the host cell cytoskeleton thereby generating cytopathic effects of infection (Lanier et al., 1996) in the secretion pathway (Figure 1). After the virus lyses, cathepsin is released into the culture supernatant and will degrade the recombinant protein. Baculovirus vectors that lack chitinase and cathepsin have thus been designed to improve the efficacy of the secretory pathway and reduce the chance of degradation of the recombinant proteins during production (BestBac 2.0, BacMagic-3, qBacII, flashBAC Gold). Other baculovirus modificactions have been constructed via the deletion of other viral replication non-essential genes, such as *p10, p24, p26* or *p74*, etc., (BacMagic-3, flashBAC Ultra); these changes also effectively improve protein yield and secretion efficiency (Figure 1). Deleting these genes reduces the unnecessary genetic burden of the recombinant virus genome and provides a more efficient baculovirus vector. Specifically, the loss of P10 increases the activity of the polyhedrin promoter. The performance of flashBAC Gold and Ultra were compared and the superior performance of Ultra cassette was demonstrated (Martinez and Targovnik, 2019).

The production of sufficient quantity and high-quality protein complexes is the prerequisite for studying their structures and functions. BEVS is particularly well suited for the simultaneous expression of multiple proteins. MultiBac is one of these systems which has been intentionally tailored to overproduce protein complexes that comprise of many subunits (Bieniossek et al., 2012). The modified baculovirus containing multiple genes could be obtained by *in vivo* Cre-LoxP fusion or Tn7R/L transposition in *E. coli* with an engineered transfer vector (Berger et al., 2004). HR-bac, a toolbox based on MultiBac, composed of a set of engineered bacmids capable of expressing a fluorescent marker to monitor virus propagation and a library of transfer vectors. This design facilitates expression screening and potential high-throughput applications (Kolesnikova et al., 2022). Another system, biGBac, is a simple, efficient and rapid baculovirus vector system that could be used to construct multigene expression. This method uses a computationally linker sequence in Gibson assembly reactions. The approach called “mix and match” allows for the generation of baculovirus genome at any assembly stage which could make up to 25 cDNAs assemble into a single recombinant baculovirus (Weissmann et al., 2016).

Another limitation in production is that viral infection causes cell lysis in the late stage. Cell lysis of baculovirus-infected cells can be delayed by expressing viral anchor proteins, such as Vankyrins, which are derived from an insect polydnavirus *Campoletis sonorensis* ichnovirus (CsIV). Baculovirus-infected Sf9 cells expressing one of the two Vankyrin proteins (P-VANK-1 or I^2^-VANK-3) showed delayed cell lysis caused by the inhibition of apoptosis, with the presence of some living cells a few days longer than those under control conditions. The anti-apoptotic effect of a Vankyrin protein is produced by regulating the immune response of host cells to viral infection (Goodin et al., 2006; Goodin et al., 2009). An important limitation of large-scale protein production by Bacmids is the easy loss of gene-of-interest (GOI) during protein expression. This instability is caused by the mini-F replicon in bacmid backbone, which is likely due to large size of gene and results in a negative selection pressure on recombinant baculovirus genomes during replication in insect cells. A latest study reported that a series of bacmids were constructed with attTn7 site displacement through lambda red genome engineering combined with SacB counterselection technology, and GOI expression at the odv-e56(pif-5) site resulted in higher expression level as compared with the original bacmid and was more stable after serial passages (Pijlman et al., 2020).

To facilitate the folding of complex/difficult target proteins, genes encoding chaperone proteins can be inserted into baculovirus vector DNA to express the chaperones at a level that is compatible with that of the overexpressed target protein. ProFold-C1 and ProFold-C2 baculovirus vector DNA can express the major human molecular chaperones Hsp40 and Hsc70 in the cytoplasm to actively fold complex target proteins (Yokoyama et al., 2000; Ngosuwan et al., 2003) ProFold-ER1 encodes the major human molecular chaperones Calreticulin and protein disulfide isomerase (PDI), which facilitate folding of target proteins in the endoplasmic reticulum (Zhang et al., 2003; Culina et al., 2004; Fourneau et al., 2004). The genes encoding for these partners are all inserted into the genome of the baculovirus distal polyhedron site.

The CRISPR-Cas9 system is a potent site-specific genome editing tool developed in recent years that has been used in many biological systems, including insect cell systems. The CRISPR-Cas9 technology offers precise genetic modification to further enhance and expand the application of the BEVS platform for recombinant protein production (Cox et al., 2015; Peng et al., 2015; Barrangou and Doudna, 2016). Based on the constructed CRISPR-Cas9 vector, the *p35* gene has been identified as an anti-apoptotic gene and a suppressor gene to the siRNA pathway (Karamipour et al., 2018). Early studies found that insect cells have an innate defense mechanism against baculoviruses that generates the secretion of inhibitors of apoptosis when the baculovirus invades (Rong et al., 2019). However, Sf9 cells stably expressing p35 protein are more resistant to apoptosis and nutritional stress, and can produce higher levels of recombinant proteins (Martinez and Targovnik, 2019). Dong et al. (2019) developed a vector called multiple editing anti-BmNPV therapeutic complex CRISPR-Cas9 system, PSL1180-Cas9-sgIE1-sgLEF11-sgGP64 (sgMultiple), that effectively regulates multiple gene editing pathways and disrupts the replication of BmNPV. This multiplex system can significantly enhance the potential of CRISPR/Cas9-based multiplex genome engineering in baculoviruses and will be beneficial in antiviral therapy. Wang et al. (2021) have also employed the CRISPR/Cas9 system to construct a recombinant baculovirus that knocks out ac106/107. They showed that ac106/107 could enter the nucleus and affect the transcription of viral RNA polymerase, which in turn could change the transcription of late genes and alter the virus proliferation. Other studies applied this system to verify the function of some genes by knockout technology (Figure 1), and found that overexpression of the Ac34 will upregulate the transcription of 38k and vp39, and Ac124 will promote the expression of chitinase, which ultimately have an effect on the replication cycle of AcMNPV (Fang et al., 2019; Zhang et al., 2019) (Table 2.

## Engineering insect cells

### Common insect cell lines

The approval of vaccines and gene therapy products for humans produced with BEVS underscores the high potential and versatility of the platform for biologics. Efforts to drive cell line engineering have occurred in response to manufacturing pressure and strict quality guidelines associated with the generation of medical supplies. Today, more than 400 kinds of cell lines from more than 100 insects have been developed for the production of baculoviruses, virus-like particles, recombinant proteins, and gene therapy vectors (Kwang et al., 2016; Liao et al., 2020). The most common cell lines sensitive to AcMNPV infection are 1) IPLB-SF21-AE (also known as Sf21) and its clonal isolate Sf9, derived from pupal ovarian tissue of *Spodoptera frugiperda*, and 2) BT1-TN5B1-4 (trade name High Five, High five) or Tn-368, derived from ovarian tissue of adults from Cabbage Looper *Trichoplusia ni* (Krammer et al., 2010) (Table 3). Fundamental differences between the cell lines must be considered with respect to the type and purpose of the protein to be produced. For instance, Sf21 and Sf9 are both highly susceptible to viral infection. The Sf9 subclone—possibly the most widely used—has a faster growth rate, higher cell density, and greater tolerance to osmosis, pH, and shear stress than Sf21 cells. High five cells, on the other hand, while being much better producers of secreted recombinant proteins as compared with Sf9 and Sf21, they release more than three times as many proteases and thus may cause higher degradation of the target protein. Sf9, Sf21 and High five cell lines are all adaptable to suspension and serum-free culture.

**TABLE 3:** Common and engineered insect cell lines.

Species	Designation	Optimization purpose	References
Spodoptera frugiperda cell line	Sf21	Experimental observation of virus titer plaque	Rong et al., (2019); Shi and Jarvis, (2007)
	Sf9	Recombinant virus amplification and packaging	Legardinier et al., (2005); Rong et al., (2019); Shi and Jarvis, (2007)
	Mimic Sf9	Glycosylation increased	Legardinier et al. (2005)
	Super Sf9	Extend cell cycle	Gomez et al. (2014)
	pIB-P-vank-1 cell line	Extend cell cycle	Goodin et al. (2006)
	pIB-I2-vank-3 cell line	Extend cell cycle	Goodin et al. (2006)
	SfSWT-1	N-glycosylation	Aumiller et al., (2003); Shi and Jarvis, (2007)
	SfSWT-3	N-glycosylation	Aumiller et al., (2003); Harrison and Jarvis, (2006); Shi and Jarvis, (2007)
	SfSWT-4	Complex N-glycans glycoproteins	Shi and Jarvis, (2007); Steele et al., (2017)
	SfSWT-5	Mammalianized N-glycosylation	Aumiller et al. (2012)
	Sfβ4GalT	Galactosylation, Sialylation	Harrison and Jarvis, (2006); Shi and Jarvis, (2007)
	Sf-RVN^Lec1^	Endo H-cleavable N-glycans.	Mabashi and Jarvis, (2021)
Trichoplusia ni cell line	BTI-Tn-5Bl-4 (High Five)	Higher levels of recombinant proteins	Krammer et al., (2010); Maghodia et al., (2020)
	BTI-Tnao38	High levels of recombinant proteins	Hashimoto et al. (2012)
	Tnms 42	Defend infection with nodule virus	Koczka et al. (2018)
	Tn‐NVN	Defend infection with nodule virus	Maghodia et al. (2020)
	Tn368	Produced more virus per cell	Scotti et al., (1996); Lynn, (2003)
Danaus plexippus	DpN1	Higher glycosylation modification	Palomares et al. (2003)
Pseudaletia unipuncta	A7S	Higher glycosylation modification	Palomares et al. (2003)
Drosophila line	DL2	Higher cell density.	Scotti et al. (1996)

A cell line derived from Sf9 cells, marketed as Express SF+, was established to produce a tri-/tetravalent influenza vaccine (Flublok, ProteinSciences Corp). A COVID-19 vaccine (Nuvaxovid, Novavax) was also developed with Sf9 cells. The divalent HPV vaccine (Cervarix, GSK) is manufactured using High five Rix4446 cells derived from *Trichoplusia ni*. Sipuleucel-T (Provenge, Dendreon Pharm.), an immunotherapy for prostate cancer, is based on a recombinant fusion protein produced in Sf21 insect cells. Other cell lines, such as Bm5, BmN, and Bme21, generated from *B. mori* embryos, and unconventional Lepidopteran cell lines, such as A7S and DpN1, from *Pseudaletia unipuncta* and *Danaus plexipus* larvae, respectively, are less commonly used.

### Improvement on glycosylation

There are two significant limitations associated with using classical insect cell lines for recombinant protein production. First, unlike the complex sialylated N-glycans produced by mammalian cells, insect cells produce fewer complex N-glycan structures, and commonly produce terminal mannose; such expression limitations may affect the stability, biological activity, or immunogenicity of the recombinant proteins. Another limitation is that cell death and lysis triggered by baculovirus infection leads to the production of immature proteins, with an increase in protease activity that negatively affects the integrity and yield of the recombinant proteins. Different approaches have been taken to overcome both these limitations and improve their performance in BEVS (Figure 1).

**FIGURE 2:**
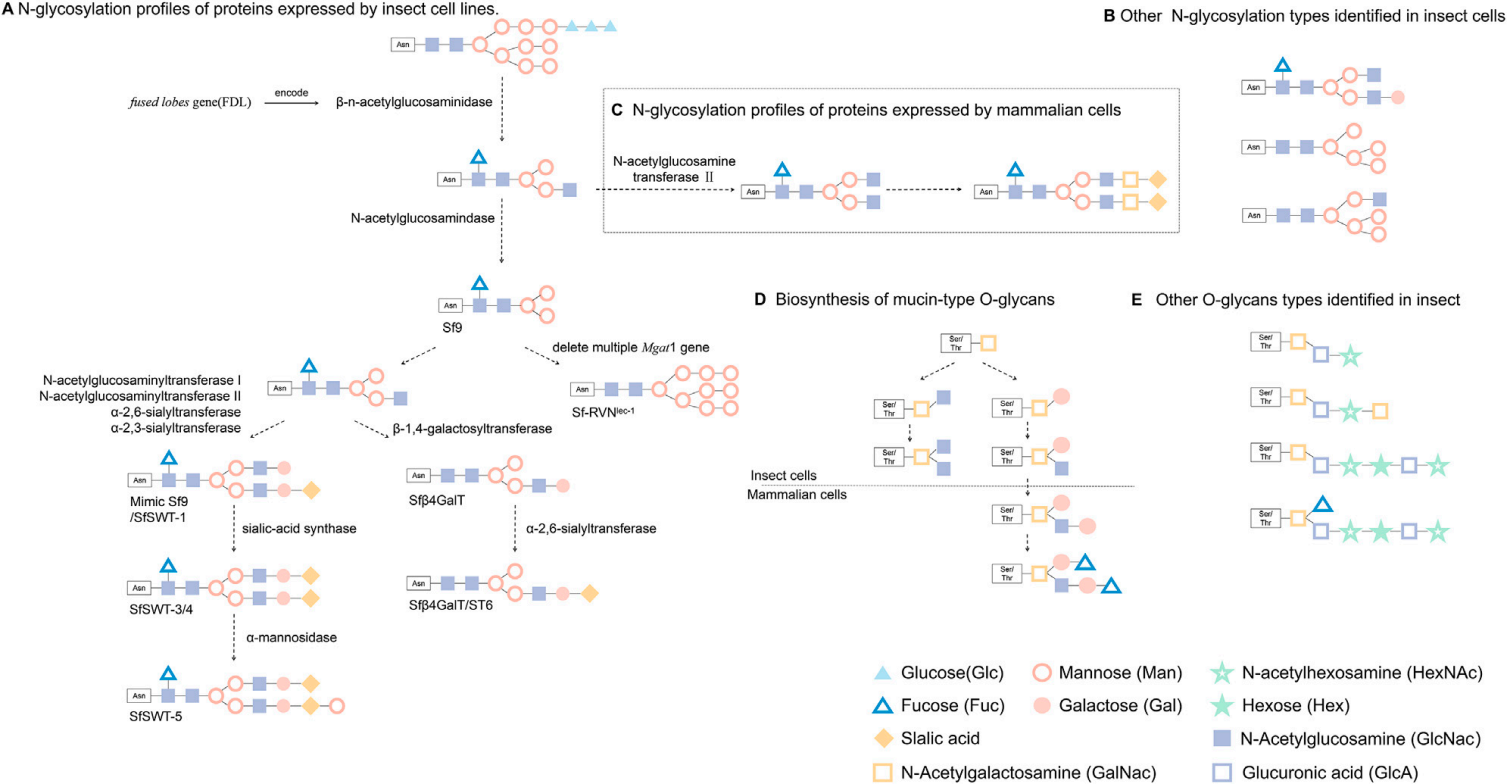
Glycosylation of foreign proteins expressed in eukaryotic cells. **(A)** Recombinant protein produced by BEVs after β-n-acetylglucosaminidase catalysis result in simple paucimannosidic structure, while some lepidopteran cell lines after cell engineering can express proteins with above N-glycosylation. **(B)** Other N-glycosylation types identified in insect cells. **(C)** Exogenous proteins produced by mammalian cells were modified to form complex N-glycans structure (This figure only shows the general steps and a common N-glycans structure.) **(D)** Biosynthetic processes of mucin-type O-glycans that have been explored in insect cells and mammalian cells. **(E)** Other common O-glycans identified in fruit fly larvae, mosquito larvae or Sf9 and High five lepidopteran cells.

Post-translational modifications (PTMs) usually happen following protein translation, which is critical for the normal biological function of proteins. Many proteins encoded by insect cell have been reported to have PTMs, including phosphorylation, glycosylation, ubiquitination, and acetylation. N-glycosylation is one of the most important post-translational modifications in eukaryotic cells. However, unlike mammalian cells, insect cells cannot add terminal galactose and sialic acid, mainly due to a lack of glycogen or glycosidase activity (Gomez et al., 2014; Palomares et al., 2021). Because BEVS cannot synthesize mammalian-type glycans, it generally hinders the platform for the broad application of glycoprotein biological products. However, insect cells have a trimming enzyme that antagonizes the elongation of paucimannose, which is not found in mammalian cells (Figure 2). Two Lepidopteran insect cell lines can transfer N-glycan precursors to nascent polypeptides and trim them to produce the same processing intermediates produced by mammalian cells. This unique processing enzyme, called a β-n-acetylglucosaminidase and encoded by *fused lobes* gene (FDL), eliminates the n-glycosyl intermediate as a substrate for N-acetylglucosamine transferase II and thereby results in simple paucimannosidic N-glycans in insect cells (Figure 2).

The mimic Sf9 (identical to SfSWT-1) insect cell line is a commercial transgenic insect cell line derived from Sf9 cells and engineered to produce highly processed, mammalian-like recombinant proteins with terminally sialylated N-glycans. Mimic Sf9 integrates with five mammalian glycosyltransferases, which causes the glycosylation level of recombinant protein to be higher than that expressed by Sf9 or High five cells (Legardinier et al., 2005). Legardinier *et al.* constructed a Sf9-derived cell line, Sfβ4GalT, which showed a high level of α-1,4-galactosyltransferase activity, and found that GP64 protein has galactosylation and sialylation when produced during baculovirus infection. Using genetic engineering to modify the expression vector, SfSWT-3 cells can be used to produce CMP-sialic acid and sialylate a recombinant glycoprotein when cultured in a serum-free medium supplemented with N-acetylman-nosamine (Aumiller et al., 2003). Aumiller *et al.* created a new transgenic insect cell line (SfSWT-5) with an inducible mammalian N-glycosylation pathway. Compared with Sf9, SfSWT-5 cells produce the same amount of recombinant protein with pronounced sialylation after doxycycline treatment (Aumiller et al., 2012). Besides these studies, others have shown that different promoters can also influence the level of glycosylation. Toth et al. (2014) found that the *39k-*inducible promoter effectively activates exogenous glycogen expression and confers higher glycoenzyme activities and human N-glycan processing efficiency as compared with the IE1 promoter. Silencing the *fdl* gene in BmN4-SID1 insect cells using RNAi technology to inhibit the expression of β-N-acetylglucosaminidase led to the production of glycoproteins with a complex-type N-glycan structure (Pena and Kamen, 2018). Mabashi *et al.* indicated the production of a functional Cas9 under IE1 promoter control, as well as functional sgRNAs under the DmU6:96Ab and BmU6-2 promoter control achieved the site-specific genome editing of the *fdl* gene using CRISPR-Cas9 in Sf9 or BmN cells (Mabashi and Jarvis, 2017). Reduced proportions of paucimannose and increased proportions of terminally GlcNAcylated structures on human erythropoietin (hEPO) were produced by polyclonal SfFDLt1 cells as compared with Sf9 cells (Mabashi and Jarvis, 2017). Masatoshi Suganuma et al. successfully generated sialylated N-glycans on proteins in silkworm by co-expression of galactosyltransferase and sialyltransferase in the presence of sialylation-related substrates being supplied during cell culture. This study introduced galactosylation and sialylation for protein expression and therefore shed light on the control of N-glycosylation in silkworm (Suganuma et al., 2018). Hiroyuki Kajiura et al. found that Bombyx mori N-acetylgalactosaminyltransferase (BmGalNAcT) is a multifunctional glycosyltransferase on N‐acetylgalactosaminylation, galactosylation, or N-acetylglucosaminylation, but overexpression of BmGalNAcT in insect cells had no effect on the major N-glycan during biosynthesis, suggested that N-glycosylation is highly regulated by the endogenous N-glycosylation machinery (Kajiura et al., 2021). Modified or transgenic cell lines are gradually being used in scientific research and will further enhance and expand the utility of BEVS as a production platform.

The heterologous proteins expression in insect cells have relatively less glycosylation profiles than that in mammalian cells (Carraway and Hull, 1989), in which N-glycosylation type dominates over O-glycosylation (Walski et al., 2017). Lopez et al. (1999) studied the glycosylation in three insect cell lines, Spodoptera frugiperda (Sf9), Mamestra brassicae (Mb) and Trichoplusia ni (Tni), and found that O-glycosylation is varied on cell types. This study demonstrated relative lower levels of galactosyltransferase and no sialyltransferase activity in insect cells, as compared to those observed in mammalian cells (Lopez et al., 1999). Stefan Gaunitz et al. (2013) firstly reported the structural characterization of phosphocholine-substituted O-glycans in Sf9. The mass spectrometry analyses showed that Sf9 oligosaccharides were consisted of short oligosaccharides (<6 residues) low in hexose (Hex) and with terminating N-acetylhexosamine (HexNAc) units, whereas Hi-5 produced a family of large O-glycans with (HexNAc-HexA-Hex) repeats and sulfate substitution on terminal residues. In both cell lines, the core N-acetylgalactosamine was dominated, but a few O-glycan cores with single fucose or hexose branches were found. In the other study, Jian Xu et al. verified a common core 1 Gal (β1-3) GalNAc disaccharide branch in the O-glycan profile without sialylation which is the major form for human proteoglycan 4 (PRG4) protein expressed in Bombyx mori (Xu et al., 2018).

### Optimization of expression

After infecting insect cells, baculoviruses would take main effect on the host cell by inducing cellular apoptosis, which in turn influences the expression time and yield of exogenous proteins that is mediated by baculoviruses. One strategy to increase production is to prolong cell survival and resist apoptosis after viral infection. RNAi technology can be applied to silence regulatory genes in the cell life cycle. For instance, when cyclin E is silenced in High five cells, cell viability is prolonged, which directly leads to an increase in the expression of the foreign protein (e.g., GFP) (Xiao et al., 2014; Pena and Kamen, 2018; Martinez and Targovnik, 2019). Dicer-2 (Dcr2) and Argonaute 2 (Ago2) genes are critical factors in response to AcMNPV infection in Sf9 cells, and their transcription level will increase after infection. Using specific dsRNA to reduce Dcr2 and Ago2 expression levels can increase the amount of viral genomic DNA, indicating that Dcr2 and Ago2 genes contribute to antiviral activity in Sf9 cells (Karamipour et al., 2018). Inserted shRNA of Caspase-1 to inhibit cell apoptosis can also significantly increase the expression of foreign proteins (Rong et al., 2019).

Steele et al. (2017) found that cell lysis could be delayed, and recombinant protein yields could be improved by using cell lines constitutively expressing vankyrin or vankyrin-encoding baculovirus vectors. Vankyrin-Enhanced Insect Cells pIB-I^2^-vank-3 cell line are transgenic insect Sf9 cells that have been engineered to stably express the *Campoletis sonorensis* ichnovirus P-vank-1 protein. Compared to regular Sf9 cells, the expression of the P-vank-1 protein prolongs the longevity of infected VE cells and thus increases recombinant protein production (Goodin et al., 2006). Other cell line engineering has used adaptive laboratory evolution (ALE) to improve the expression of recombinant foreign proteins: this involves adapting cells to grow efficiently under non-standard culture conditions. For example, using ALE methodology such as a high-pH, a novel insect cell variant (SfBasic) was adapted to maintain cell growth in the pH range of 7.0–7.2. Using these cells, Chikungunya VLP yields were produced up to 11 times higher than that in normal cells (Wagner et al., 2014). In addition, Fernandes et al. (2020) reported culturing hypothermic-adapted Sf9 and High five cells at 22°C instead of 27°C, which increased the expression of HIV-Gag by 26-fold (Sf9 cells) and 10-fold (*T. ni* cells) as compared with their non-adapted counterparts (Cox, 2021).

## Conclusion and perspectives

The BEVS has extensive utility in biological research, in terms of gene therapy, foreign protein expression, and for the screening of candidate vaccine molecules for medical research. The current rapid development in molecular biotechnology tools make this an ideal time to expand upon the application and advantages of the insect cell-baculovirus expression platform. Indeed, engineering genome composition, and optimizing protein stability, yield and the production of appropriate posttranslational modifications will provide diverse applications for BEVS in the near future. For example, the expression level of heterologous proteins could be improved by elevating the recombination efficiency, or by re-combination of expression elements or deleting non-essential components of baculovirus elements, or using RNAi technology or CRISPR Cas9 to edit the baculovirus vector to gain anti-apoptotic ability and therefore prolong the expression process. Then, the stability of recombinant bacmid in scale-up production should be underscored and considered over the recombination efficiency and integration site of GOI. In addition, the genome of host insect cell lines could also be engineered to extend the lifespan or to strengthen its post-translational modifications, especially for the mammalian cell-like N-glycosylation profiles. We believe that the current fast and advanced BEVS technology may continue to forge a solid impact in biomedical applications.
